# The relationship between acculturation and mental health of 1st generation immigrant youth in a representative school survey: does gender matter?

**DOI:** 10.1186/s13034-020-00334-6

**Published:** 2020-07-17

**Authors:** Eva M. Klein, Kai W. Müller, Klaus Wölfling, Michael Dreier, Mareike Ernst, Manfred E. Beutel

**Affiliations:** Department of Psychosomatic Medicine and Psychotherapy, University Medical Center of the Johannes Gutenberg University Mainz, Langenbeckstraße 1, 55131 Mainz, Germany

**Keywords:** Migration, Immigrant youth, Acculturation, Mental health, Gender differences

## Abstract

**Background:**

Although gender plays a pivotal role in the psychological adaptation of immigrant youth, its association with acculturation strategy and mental health among 1st generation immigrant adolescents are still scarce and inconsistent. Therefore, the purpose of the current study was to investigate gender-related differences in acculturation patterns and their association with mental health (internalizing and externalizing problems).

**Methods:**

Self-reported data of immigrant adolescents (*N *= 440) aged between 12 and 19 years (*M *= 16.2; *SD *= 1.6) was collected in a representative German school survey. Fifty-one percent of the sample were female (*n *= 224). Almost half of the sample was born in the Former Soviet Union, followed by Poland (9.3%). Sociodemographic variables, acculturation strategies, and internalizing as well as externalizing problems were assessed by questionnaires.

**Results:**

Confirmatory factor analysis supported the four- dimensional model of acculturation styles (assimilation, integration, separation and marginalization). Whereas girls more often showed an integration pattern, boys scored higher on the separation and marginalization scale. After adjusting for age and educational level, regression analyses revealed for both gender that marginalization was associated with more internalizing problems. Separation was related to more externalizing problems.

**Conclusion:**

1st generation adolescents experiencing a lack of belongingness to German society, socio-economic and educational disadvantages might be particularly vulnerable to mental distress. Findings are discussed in terms of gender-related differential socialization processes in context of immigration.

## Background

In many societies worldwide, the proportion of immigrant youth is steadily growing. According to Federal census statistics [1], the proportion of immigrants in the German population under 18 years has risen to about 36% in 2017. As immigrant youth are confronted with various challenges affecting their mental health like acculturative stress and social disadvantages [2], promotion of their psychological and sociocultural adaptation has become a central social and political issue. In addition to accomplish age-salient developmental tasks like their nonimmigrant peers, immigrant youth need to master acculturative tasks such as the adaptation to a new cultural environment [3, 4]. Although there is evidence that the majority of young immigrants adapt well in their new intercultural setting [5], research has consistently demonstrated that immigrant adolescents are at increased risk for psychological distress [6–9], which is particularly the case for 1st generation immigrants [10–12]. Consistent with the literature, we found in our previous analysis of a representative school survey with 8518 pupils aged 12 to 19 years attending various school types differences in sociocultural and psychological adaptation between adolescents with migration background and their majority peers [13]. The study showed that 1st generation immigrants scored higher on internalizing and externalizing problems than 2nd generation immigrants or non-immigrants. The differences, however, were small. Regarding sociocultural adaptation, 1st generation immigrants experienced more educational disadvantages (e.g. more frequent grade repetitions). Regardless of immigration background, female adolescents reported more internalizing problems than their male counterparts. No gender difference emerged for externalizing problems [13].

## Mental health, acculturation and gender

In order to cope with acculturative stress, different acculturation patterns, describing beliefs, attitudes and behaviors related to the culture of origin and the host culture, are applied. On the basis of the bi-dimensional model, Berry [14] proposed four acculturation strategies: *Integration* describes a strong involvement in both the culture of origin and the host culture. A*ssimilation* refers to a strong orientation to the culture of the receiving society combined with low maintenance of the heritage culture. *Separation* is characterized by strong maintenance of the heritage culture and limited intercultural contact to the host society. *Marginalization* is defined by low involvement in both cultures. Previous research demonstrated that immigrant youths’ mental health is related to their acculturation strategy [15]. Integrated acculturative patterns have been identified as protective factors for psychological adaptation, while marginalization was associated with poor health outcomes [7, 15, 16]. For example, in a recent study of 416 adolescents (*M*_*age*_= 16.07, *SD*_*age*_= 2.90; 71% immigrants) Kupper et al. [17] found higher levels of depressive symptoms among immigrants compared to their non-immigrant peers. Symptom burden was in turn related to participants’ acculturation pattern. Immigrants who felt more strongly oriented towards the host culture reported less severe levels of depression. Orientation towards the culture of origin, however, was not related to the depression score. Additionally, the study suggested a gender effect for the strength of the relationship: the negative relation between the orientation towards host culture and depressive symptoms was more pronounced for boys than for girls. Likewise, previous research has recognized the pivotal role of gender in the relationship between immigrant youths’ mental health and acculturation orientation [18]. In immigrant youth, there is evidence that girls showed better sociocultural adaption (e.g. school success) while boys had better psychological adjustment [15, 19]. Regarding acculturation orientation, some studies found no gender differences [17, 18]. In contrast, other studies have provided evidence that girls more often reported an integrated acculturation pattern and a stronger involvement in the host culture, while boys were more orientated towards their ethnic culture of origin or showed an inconsistent acculturation orientation [15, 19]. Studies have suggested that for girls, factors indicating social integration like social support [20] or school connectedness [18] were important predictors of their mental well-being. As mentioned above, acculturation experiences seemed to play a more salient role for boys’ mental health status [17, 20]. As the majority of studies explored internalizing problems as indicators for mental health, much less is known about the relationship between acculturation style, gender and externalizing symptoms. Externalizing problems like conduct problems, however, are highly prevalent among adolescents [21, 22] and thus important to assess within a sample of this age group.

### The current study

The purpose of the current study is to deepen the understanding of the mechanism underlying our previous empirical finding which indicated reduced mental health of 1st generation immigrants [13]. Our data set gave us the novel opportunity to explore the associations between acculturation strategy, mental health and gender among 1st generation immigrant adolescents using self-reported data of a representative school survey. Beyond exploring internalizing problems, this study contributes to the existing research by additionally including externalizing problems as a common form of manifestation of distress in adolescents. The following research questions are addressed:

1. How do 1st generation immigrant girls differ from 1st generation immigrant boys in regard to their acculturation strategy?

2. How are acculturation strategies associated with mental health (internalizing and externalizing problems) among girls and boys after adjusting for age and educational level?

Based on theoretical assumptions and empirical evidence from previous research, we assumed that separation and marginalization are associated with reduced mental health, while integration is a protective factor against distress. Due to previous inconsistent findings, we examined differences between girls and boys regarding acculturation strategy at an exploratory level. It was hypothesized that the strength of the relationship between acculturation strategy and mental health may differ between girls and boys, with a stronger impact of acculturation difficulties on boys’ mental health.

## Methods

### Sample and procedure

The current study is based on a subsample of 1st generation immigrants participating in a representative school survey. Self-reported data were collected in randomly selected schools stratified by school type (secondary school, intermediate secondary school, comprehensive school, grammar school, vocational school), school grade and area selected by number of inhabitants (< 10,000 inhabitants, > 10,000 and < 100,000 inhabitants, > 100,000 inhabitants) in North Rhine-Westphalia and Rhineland Palatinate in 2012. Each participant who provided informed consent completed the questionnaires in their schools in the presence of a trained investigator. For participants aged under 14 years an additional informed consent of their parents was required.

The study design, procedure and applied questionnaires were approved by the institutional ethics review boards. Three positive ethical votes were required and received. The relevant ethical chambers of Rhineland-Palatinate (Mainz, Germany) and both ethical chambers for North Rhine-Westphalia, specifically the ethical chamber Westfalen-Lippe (Münster, Germany) and the ethical chamber of North Rhine (Düsseldorf, Germany) voted positively.

*N *= 440 1st generation immigrants were analyzed in the current study. Participants with missing data on the acculturation scale (n = 57) were not considered in the analyses. Dropout analyses revealed that participants with missing values did not differ from the analyzed sample regarding gender, age, country of origin and mental health. 2nd generation immigrants were prior excluded from the study due to anticipated different socialization processes.

### Questionnaires

Migration status was defined according to the German micro census (1). Participants who had migrated themselves to Germany and those who had at least one parent born abroad were considered as 1st generation immigrants. The country of origin of their mothers and fathers was also assessed.

The Strengths and Difficulties Questionnaire [23] is a brief screening questionnaire measuring emotional and behavioral problems. The internationally well-established scale has been evaluated in research and clinical practice [24]. A recent study by Runge and Soellner [25] suggested the validity of the SDQ as screening tools in immigrant children. The subscales internalizing problems (IP) and externalizing problems (EP) with eight items each were assessed [26]. Internalizing problems comprised anxiety symptoms and depressed mood, while externalizing problems covered conduct problems like impulsivity, aggression and hyperactivity. The items were rated on a 3-point scale (1 = “not true”, 2 = “somewhat true”, 3 = “certainly true”). Values of Cronbach’s alpha were acceptable in the current study (Cronbach’s alpha IP = .71; Cronbach’s alpha EP = .66).

The *Scale for Measuring Applied Acculturation Strategy* [27] was applied for measuring the orientation towards both the receiving and heritage cultures. Each acculturation style (assimilation, integration, separation and marginalization) was assessed with three items each covering language use, social contacts, values and attitudes. Participants rated the items on a Likert-scale ranging from 1 = “not at all true” to 4 = “totally true”. The internal consistency in the current study was good for the separation scale (Cronbach’s alpha = .79), acceptable for the assimilation scale (Cronbach’s alpha = .68), integration scale (Cronbach’s alpha = .68), and marginalization scale (Cronbach’s alpha = .61).

### Statistics

For group comparisons between girls and boys χ^2^-tests for categorical variables were calculated. To analyze gender differences an ANCOVA was used with internalizing problems and externalizing problems as dependent variables, respectively a MANCOVA was calculated with assimilation, integration, separation and marginalization as dependent variables. Before analyzing the acculturation styles, a confirmatory factor analysis (CFA) was calculated first evaluating the suggested four-factor solution of the acculturation scale. The model was estimated with the maximum likelihood method approach. Model fit was evaluated by using following model fit indices [28]: Chi square statistic; the comparative-fit-index (CFI) and the Tucker-Lewis Index (TLI) to describe incremental fit; the root means square error of approximation (RMSEA) was used as an absolute measure of fit. Values of TLI and CFI close to .95 or higher indicate a better fit. RMSEA should be 0.08 or smaller. Multiple linear regression analyses with internalizing problems, respectively externalizing problems as dependent variables and gender and acculturation styles as independent variables were conducted. Interactions terms (gender*acculturation style) were added in the model. Sensitivity analysis was performed according to Soper [29]. A minimum sample of 98 participants was required in order to test a regression model with 12 predictors to observe a small effect (*f*^*2*^= .02) with a statistical power level of .08. Hence, the current sample size was sufficient to carry out the analyses. All analyses considered age and higher education (i.e. grammar school) as covariates. Data analyses were performed by SPSS Version 21.0 and AMOS© 21.0.

## Results

Participants were aged between 12 and 19 years (*M *= 16.2; *SD *= 1.6). 50.9% of the sample were female (*n *= 224). About half of the sample were from countries of the Former Soviet Union (52.5%), followed by Poland (8.4%), Arabic-Islamic countries (7.7%), Turkey (6.6%), Former Yugoslavia (5.9%) and countries in Middle- and West Europe (4.5%). 14.3% were from other countries. The four largest groups of country of origin were compared regarding mental health and acculturation controlling for age and education. There were no group differences regarding internalizing problems (*F*(3, 330) = 1.36; *p *= .27) and acculturation pattern (*F*(4, 330) = 1.09; *p *= .36). However, the groups differed with respect to externalizing problems (*F*(3, 330) = 3.19; *p *= .024; *η*^*2*^= .03). The highest levels of externalizing problems were found among participants from Turkey (*M *= 6.62; *SD *= 3.07), followed by participants from Arabic-Islamic countries (*M *= 5.79; *SD *= 2.67), Poland (*M *= 5.75; *SD *= 2.67) and Former Soviet Union (*M *= 4.91; *SD *= 2.94).

The distribution of the countries of origin was comparable among girls and boys. No differences emerged for age and grade repetition, but for school type. Whereas more girls attended grammar school, more boys attended vocational school. After controlling for the covariates, girls reported higher levels of internalizing problems than boys. Age was a significate covariate (*F*(1, 349) = 4.89; *p *= .03; *η*^*2*^= .01) and was correlated positively with internalizing problems. The ANCOVA with externalizing problems as outcome variable revealed no gender differences, but both covariates were significant *(F*_age_(1, 439) = 12.43; *p *< .001; η^2^ = .03; *F*_grammar school_(1, 439) = 6.93; *p *= .009; η^2^ = .02). The results are shown in Table 1.

**Table 1:** Sample description

	Girls	Boys	Total	Test statistics
*N* (%)	224 (50.9)	216 (49.1)	440	
Age	16.18 (1.57)	16.28 (1.61)	16.23 (1.59)	*ns*
School type				*χ*^*2*^ (4. *N *= 440) = 13.23; *p *= .01; *Cramer’s* *V *= .17
Secondary school	17.9	15.7	16.8	
Intermediate secondary school	18.8	16.2	17.5	
Comprehensive school	11.6	6.9	9.3	
Grammar school	22.8	16.2	19.5	
Vocational school	29.0	44.9	36.8	
Grade repetition (yes)	36.1	38.3	37.2	*ns*
Country of origin				*ns*
Former Soviet Union	50.5	54.5	52.5	
Poland	9.3	7.6	8.4	
Arabic-Islamic countries	6.9	8.5	7.7	
Turkey	6.9	6.3	6.6	
Former Yugoslavia	6.9	4.9	5.9	
Middle- and West Europe	4.6	4.5	4.5	
Other	7.3	7.0	14.3	
Mental health				
Internalization problems	5.08 (3.11)	3.46 (2.61)	4.29 (2.99)	*F*(1. 439) = 35.86; *p *= .000; *η*^*2*^ = .08
Externalization problems	5.22 (2.93)	5.19 (2.98)	5.21 (2.95)	*ns*

Percentages are presented for categorical variables; *M (SD)* are presented for continuous variables

Confirmatory factor analysis revealed that both incremental fit indexes (CFI = .94; TLI = .92) and absolute measures of fit indexes were good (RMSEA = .05) supporting the four- dimensional model (χ^2^ (48, *N *= 440) = 115.5, *p *< .001). Two CFA separated for boys and girls were calculated (boys: (χ^2^ (48, *N *= 216) = 86.13, *p *< .001); CFI = .94, TLI = .92, RMSEA = .06; girls: (χ^2^ (48, *N *= 224) = 113.5, *p *< .000); CFI = .83, TLI = .86, RMSEA = .07). The fit indices suggested a slightly better model fit within the boys’ group. Within the girls’ group, only the RMSEA suggested an acceptable model fit. Detailed psychometric characteristics of the acculturation scale, separated for girls and boys are provided in Appendix.

**Fig. 1:**
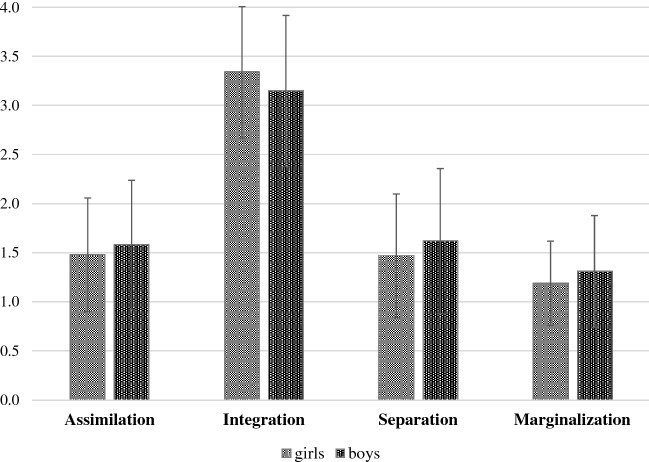
Acculturation styles separated for girls and boys. Presented are means and standard error for each acculturation style

Figure 1 presents the mean values of each acculturation style separately for girls and boys. Integration was the acculturation style with the highest mean values, followed by assimilation and separation with similar mean scores. The means for marginalization were the lowest. The results of the MANOVA revealed a main effect for gender differences in acculturation style (*F*(4, 433) = 3.79; *p *= .005; *η*^2^ = .03). Girls more often showed an integration pattern (*F*(1, 436) = 7.23; *p *< .007; *η*^*2*^= .02) than boys. Boys scored higher on the separation scale (*F*(1, 436) = 4.88; *p *= .028; *η*^*2*^= .01) and on the marginalization scale (*F*(1, 436) = 6.04; *p *= .014; *η*^*2*^= .01) than girls. The covariate age was significant (*F*(4, 433) = 7.11; *p *< .000; *η*^*2*^= .06). Older adolescents reported higher values of assimilation (*F*(1, 436) = 6.84; *p *= .009; *η*^*2*^= .02) and integration (*F*(1, 436) = 12.39; *p *< .000; *η*^*2*^= .03), but lower values of separation (*F*(1, 436) = 8.08; *p *= .005; *η*^*2*^= .02). There was a main effect of school type (*F*(4, 433) = 4.71; *p *= .001; *η*^*2*^= .04) with adolescents attending a grammar school reporting lower scores for the separation scale (*F*(1, 436) = 9.51; *p *= .002; *η*^*2*^= .02).

The multiple linear regression analyses (Table 2) yielded significant results for both the model with internalizing problems as dependent variable (*F*(11, 439) = 6.54; *p *= .000; *R*^*2*^= .12) and the model with externalizing problems as dependent variable (*F*(11, 439) = 5.35; *p *= .000; *R*^*2*^= .10). Internalizing problems were associated with female gender and higher age, whereas externalizing problems were associated with younger age and not attending grammar school. Regarding acculturation style, marginalization was related to higher scores in internalizing problems. Higher levels of separation were associated with more externalizing problems. Interaction effects between gender and acculturation styles were not found either for internalizing problems nor externalizing problems.

**Table 2:** Separate regression analyses of internalization problems and externalization problems (*N *= 440)

	Internalization problems				Externalization problems		
	*β*	*T*	*p*		*β*	*t*	*p*
Gender	.31	6.70	*.00*		.05	1.11	.27
Age	.10	2.06	*.04*		− .14	− 2.87	*.00*
Gymasium (yes)	.02	.38	.70		− .08	− 1.75	.08
Assimilation	.10	1.59	.11		− .00	− .49	.96
Integration	− .04	− .68	.50		− .00	− .62	.95
Separation	− .00	− .05	.96		.26	4.03	*.00*
Marginalization	.20	3.18	.*00*		.10	1.53	.13
Gender * assimilation	− .03	− .53	.60		− .01	− .14	.89
Gender * integration	.08	1.27	.20		− .02	− .31	.76
Gender * separation	.05	.86	.39		− .06	− .93	.35
Gender * marginalization	− .00	− .05	.96		.06	1.05	.30

Male = 0; female = 1

## Discussion

The current study aimed to deepen the understanding of former findings suggesting elevated prevalence of internalizing problems and externalizing problems among adolescent 1st generation immigrants by exploring the role of acculturation strategies considering potential gender differences. The findings of this school-based survey suggested that girls and boys differed in the prevalence of internalizing problems and their acculturation pattern. Regardless of gender, marginalization was associated with more internalizing problems, whereas a separated acculturation pattern was linked to more externalizing problems.

### Acculturation styles

The results of the confirmatory factor analyses supported the suggested model by Berry [14] with four different acculturation strategies (assimilation, integration, separation and marginalization). The integrative acculturation pattern was most commonly represented among the adolescents, followed by assimilation and separation. Marginalization was the acculturation style with the lowest approval rate. This finding is consistent with previous research on acculturation patterns in immigrants indicating that integrating language use, social contacts, and values of both the host cultural and cultural of origin was the most common acculturation pattern, whereas low involvement in both cultures was the least prevalent acculturation style [15, 17, 30, 31]. Gender-related pattern of acculturation styles were found supporting previous studies [15, 19] with boys scoring higher on separation and marginalization than girls. In contrast, girls more often showed an integrative acculturation pattern compared to boys. The specific gender differences in acculturation profiles might be explained by gender-related different socialization processes and experiences. Girls particularly tend to cherish interpersonal involvement and social ties (cf. [32]). Therefore, girls are more likely to establish a new social network with peers in the receiving society, while remaining attached to peers and family members in the country of origin. Boys, however, more frequently reported a main orientation towards their peers, culture and attitudes of their country of origin and a low involvement both cultures. One explanation for this finding might be that boys are more prone to experience discrimination [20, 33]. Adolescents experiencing discrimination are more likely to reject close contact with the receiving society and be more orientated towards their own cultural group [15] in order to avoid feelings of exclusion and protect their self-esteem. Indeed, previous studies showed that perceived discrimination was related to separation and marginalization patterns [15, 31]. In a sample of 170 immigrants from the former Soviet Union aged 12 to 19 years, Jasinskaja-Lahti and Liebkind [34] found that the more adolescent immigrants adhered to traditional family values promoting their relationship to their parents, the less contact was made with the receiving society which seemed to protect them against perceived discrimination. This observed connection was particularly salient among younger males compared to their female peers. An alternative explanation might be that particularly among adolescents from traditional cultures, girls are more motivated to attach to “Western” values that give women greater freedom [35], whereas boys are more likely to identify themselves with traditional values like masculinity norms in order to maintain their social status [13]. Moreover, the results showed that adolescents were more likely to report an assimilation and integration acculturation profile with increasing age, whereas the separation acculturation profile was more likely among younger pupils. This might be explained from a developmental perspective contending that the societal influence of peers and contexts outside of the family such as the school environment become more prominent whereas the contact to one’s parents becomes less frequent [32].

### Acculturation styles and mental health

As expected the findings of the current study revealed a significant correlation between separation and marginalization and reduced mental health. Regarding gender differences, more girls reported internalizing symptoms compared to boys. Higher prevalence of depression and anxiety among adolescent girls has often been demonstrated in research on adolescent’s psychopathology [9, 36] and across immigrant adolescences [8, 11, 18, 20, 37, 38]. In contrast to our hypothesis, we found no gender-related pattern in the association between acculturation and mental health. Hence, the findings suggest that regardless of gender, marginalization was related to higher levels of internalizing problems like anxiety and depressiveness. This corroborates previous studies identifying marginalization as the least adaptive pattern compromising well-being [14, 17, 35, 39]. Weak bonding to the members of the host culture and the culture of origin might create social isolation and ambivalence in the process of identity development which can result in depressive and anxious symptoms. Additionally, our earlier findings demonstrated that marginalization was related to self-insecurity, which is a core symptom of anxiety disorder [40]. However, due to the cross-sectional study design, the causal direction of the association between marginalization and internalizing problems cannot be determined, as depression and anxiety might also facilitate an acculturation pattern of marginalization.

Separation as an acculturation style was related to externalizing problems like hyperactivity and conduct problems. This finding contradicts the hypothesis that strong identification with the heritage culture might create a feeling of belongingness and buffer the harmful effect of perceived discrimination with positive impact on individual’s mental well-being [35]. The association between separation and diminished mental well-being rather supports the notion that separation might be a consequence of rejection and prejudice from the host society, deepening the perceived gap in social norms and acculturation attitudes between members of the dominant culture and minority group members. In consequence, immigrants’ distress and fears not to be socially accepted as a full member in the new society might increase [20, 35], which might be expressed in behavioral problems. Despite inconsistent finding in acculturation research, empirical evidence demonstrated the association between a separation profile and reduced physical and mental health in immigrants living in Germany [41, 42]. The educational disadvantages of immigrants compared to their not immigrated peers with German origin [13] might further represent a potential threat to immigrants not to achieve social status in the majority society [35], which might be buffered by the identification with the culture of origin. Following this hypothesis, the lower educational attainment in immigrant boys might explain their higher separation profiles. However, school engagement, which has been identified as protective factor for adolescents’ mental health, requires a high orientation towards the receiving culture [8].

In contrast to our hypotheses and previous research, we could not identify integration as protective acculturation pattern. Nguyen and Benet-Martínez [43] concluded from a meta-analysis on the association between biculturalism and adjustment that varying results might be explained by different acculturation measures, sample characteristics and diverse life domains outcomes. In addition, both age and education level must be taken into account, given their impact on acculturation styles and mental health status.

### Limitations and future research

Finally, several limitations of the current study need to be considered while interpreting the results. The data obtained was based on self-report, which can lead to response bias. In regard to the sample, the large proportion of adolescents from the Former Soviet Union is probably explained by historical reasons as the majority of these immigrants are very likely so-called “Spätaussiedler” (a German minority who had been living in the former Soviet Union and other former eastern bloc states and re-migrated to Germany). Despite the applied procedure of random selection, the proportions of the immigrants’ countries of origin is not according to representative data of the population and might reflect local clustering (as not all German federal states were included). While we took effort to recruit a representative sample, we cannot preclude that certain groups of migrants (e.g. Turkish immigrants) were underrepresented. Importantly, after the migration movements starting in 2015, the proportion of immigrants from Arabic-Islamic countries could be higher today compared to 2012 when the survey was conducted.

A further significant concern is participation bias given that the sample was limited to immigrants with sufficient language skills as only German questionnaires were utilized. Language skills determine the intensity of interpersonal contact in the receiving society [44] and are key factors for acculturation. Given the association between lower linguistic proficiency in the new language and separation as acculturation strategy [45], the separation profile may have been underrepresented in the current sample. Further, immigrants who participated in the study might be heterogeneous in terms of pre-migration experiences, causes of migration and migration trajectories. Methodological limitations refer to the moderate internal consistency of the marginalization subscale, which is particularly the case for girls, and the externalizing problems scale of the SDQ with the latter having already been reported in previous studies [21]. Literature on acculturation research [46] pointed to the complexity of acculturation processes, constituting a methodological challenge to operationalize acculturation patterns. There has also been a growing awareness of the need to empirically investigate measurement invariance as a prerequisite for group comparisons. As this methodological issue was beyond the scope of the present paper, we could not address it, but it should be a motive for future research. These limitations have to kept in mind while interpreting the current results and therefore replication studies are required.

Moreover, education level was used as an indicator for socioeconomic status. Despite its significant correlation with migration status, socioeconomic status is difficult to assess in youth samples due to lacking information from parents. However, the representative sampling in the current study promoted variation in socioeconomic status across the sample. As mentioned above, the cross-sectional study design does not allow to draw causal conclusions. Longitudinal studies are required to examine developmental trajectories of acculturation pattern and mental health across adolescents. Since we did not assess age-salient developmental tasks in adolescence like developing independence from parents, we could not disentangle the impact of acculturation stress and general challenges in puberty on immigrated adolescents’ mental health. To develop a full picture of immigrant youth adaptation, more studies will be needed that additionally take family-related factors into account like parental support (cf. 4) and parental acculturation styles, as well as contextual factors like perceived discrimination, acculturation orientation and multiculturalism in the receiving society.

## Conclusion

Consistent with previous findings of this school-based survey, gender-related considerations within the acculturative process are pivotal to understand young immigrants’ psychological adaption. 1^st^ generation adolescents experiencing socio-economic hardship, educational disadvantage and a lack of belongingness to the host society might be especially vulnerable to mental distress. The results have practical applications suggesting that e.g. promoting school engagement in immigrant pupils by teachers and through means of policy can be useful to reduce separation and marginalization as risk factors for distress. Although we were not able to examine the mechanisms underlying the association between acculturation styles and mental health, differentiating internalizing and externalizing problems might be useful for future studies in order to identify possibly unique patterns shaping different syndromes in adolescence.

## Data Availability

The datasets used during the current study are available from the corresponding author on reasonable request.

## Appendix: Psychometric characteristics of the acculturation scale

**Table:**

	Total (n = 440)			Girls (n = 224)			Boys (n = 216)		
	*M*	SD	*rIT*	*M*	*SD*	*rIT*	*M*	*SD*	*rIT*
Item 1	1.81	.97	.51	1.76	.94	.45	1.87	.99	.56
Item 2	1.33	.64	.54	1.29	.57	.51	1.38	.69	.55
Item 3	1.45	.76	.47	1.40	.72	.44	1.50	.80	.48
Item 4	3.10	1.04	.44	3.14	1.04	.44	3.05	1.04	.45
Item 5	3.59	.78	.52	3.70	.68	.52	3.48	.86	.50
Item 6	3.08	.96	.53	3.2	.87	.48	2.94	1.04	.56
Item 7	1.74	.95	.65	1.65	.87	.69	1.84	1.03	.61
Item 8	1.44	.74	.65	1.37	.69	.59	1.52	.78	.68
Item 9	1.46	.77	.61	1.40	.72	.52	1.52	.82	.67
Item 10	1.40	.84	.42	1.31	.72	.42	1.50	.93	.40
Item 11	1.15	.50	.42	1.10	.41	.30	1.19	.58	.47
Item 12	1.21	.64	.48	1.17	.59	.43	1.25	.69	.50
Cronbachs alpha									
Assimilation	.68			.64			.70		
Integration	.68			.66			.69		
Separation	.79			.76			.79		
Marginalization	.61			.56			.63		

rIT: corrected item correlation

## Abbreviations

EP: Externalizing problems
IP: Internalizing problems
SDQ: Strengths and Difficulties Questionnaire
